# Functional outcomes and complications following salvage radical prostatectomy for post radiotherapy recurrent prostate cancer: A meta-analysis

**DOI:** 10.1097/MD.0000000000044440

**Published:** 2025-09-26

**Authors:** Yachun Zhou, Xiujun He, Qin Yu, Qing Zhong

**Affiliations:** a Health Management Center, Beilun People’s Hospital, Ningbo, China; b Nursing Department, Beilun People’s Hospital, Ningbo, China; c Department of Urology, Beilun People’s Hospital, Ningbo, China.

**Keywords:** complications, functional outcomes, prostate cancer, radical prostatectomy, radio-recurrent, salvage

## Abstract

**Background:**

Numerous patients with radio-recurrent prostate cancer are treated with salvage open radical prostatectomy (RP) or robot-assisted RP. It’s important to understand the side effects of these treatments. This study aimed to explore the rates and types of treatment-related adverse events.

**Methods:**

A thorough review of PubMed and EMBASE databases up to September 2024 was conducted, focusing on severe complications and functional outcomes at 1-year after salvage surgery. Severe complications were assessed using the Clavien–Dindo scale. Functional outcomes included urinary incontinence and erectile dysfunction (ED).

**Results:**

A total of 34 studies were included, comprising 28 investigations on serious complications and 20 studies focused on functional outcomes. The overall incidence of serious complications was 14.56% (95% confidence interval [CI]: 13.44–15.74%), with the highest rates observed for urethral stricture (5.90%, 95% CI: 4.23–7.98%). One year post-salvage surgery, the incidence of any-grade urinary incontinence (≥1 pad/d), moderate to severe incontinence (≥2 pads/d), and severe incontinence (≥3 pads/d) was 49.57%, 24.62%, and 17.75%, respectively. Additionally, the incidence of ED following salvage RP at 1-year was 73.21%. There were no significant differences in the incidence of serious complications and ED between open and robotic surgery. However, the incidence of moderate to severe incontinence was lower in the robotic group (open vs robotic (43.36% vs 31.39%), odds ratio (OR; 95% CI) = 1.38 (1.14–1.68), *P* = .001). Patients with longer operative time had significantly higher rates of severe complications (≥150 minutes vs <150 minutes (16.85% vs 3.55%), OR 4.75 (95% CI: 2.92–7.71), *P* < .001) and moderate to severe urinary incontinence (≥150 minutes vs <150 minutes (40.31% vs 26.99%), OR 1.49 (95% CI: 1.17–1.90), *P* < .001).

**Conclusion:**

For radio-recurrent prostate cancer, salvage surgery should be performed with caution and only under strict indications due to the relatively high incidence of adverse events. Robotic surgery and shorter operative times are associated with a lower incidence of moderate to severe urinary incontinence, while longer operative times are linked to an increased risk of severe complications.

## 1. Introduction

Prostate cancer (PCa) is the most common visceral malignancy in men in the United States.^[1]^ About 1 quarter of men with newly diagnosed localized PCa preferred nonsurgical local treatment.^[2]^ In the case of primary radiotherapy, 41.0% to 73.8% of men will develop biochemical recurrence within 10 years.^[3–5]^ Local salvage therapy is not an option for most patients, with androgen deprivation therapy (ADT) or watchful waiting being their preferred options.^[6,7]^ However, as a systemic therapy, ADT is associated with a number of toxic side effects^[8]^ such as cardiovascular toxicity^[9]^ and osteoporosis.^[10]^ The optimal local salvage therapy for recurrent PCa after initial radical radiotherapy remains controversial. Current local salvage treatment options include salvage radical prostatectomy (SRP), brachytherapy (BT; low-dose-rate brachytherapy, or high-dose-rate (HDR) brachytherapy), stereotactic radiotherapy, high-intensity focused ultrasound and cryotherapy. Previous meta-analyses have shown no significant difference in tumor control outcomes between SRP and nonsurgical salvage modalities.^[11,12]^ However, the nonsurgical salvage options appeared to have a less severe toxic outcome compared to SRP.^[11,12]^ Therefore, only few patients with BCR after radiotherapy will receive SRP as salvage therapy.^[13]^ In recent years salvage robot-assisted radical prostatectomy (SRARP) has emerged as an alternative to salvage open radical prostatectomy (SORP) for these patients. Studies suggest that SRARP is a feasible treatment option for recurrent PCa patients who meet certain criteria after initial treatment, with similar oncologic outcomes and shorter recovery times compared to SORP. The incidence of complications appears to be lower in the SRARP group, but there is little assessment of functional outcomes in the literature.^[14–17]^

Although previous reviews have summarized and compared various salvage options,^[11,12,18]^ there are no systematic statistics on complications and functional outcomes of SRP and a comparison of open versus robotic surgery. Therefore, the aim of this study is to evaluate the complications of SRP and their impact on urinary incontinence and sexual function outcomes. In addition, we will compare the complications and functional outcomes of SORP and SRARP.

## 2. Materials and methods

### 2.1. Research design

Our meta-analysis adhered to the Preferred Reporting Items for Systematic Reviews and Meta-Analyses guidelines. The evaluation protocol was prospectively registered in the International Prospective Register of Systematic Reviews and is publicly accessible under registration number CRD42024593898.

### 2.2. Data source and searches

We conducted a meticulous and systematic literature search across 2 reputable electronic databases, Embase and PubMed, spanning articles from their inception to September 30, 2024. Full-text articles were independently screened for eligibility by 2 investigators. The search strategy utilized relevant terms: (radiation therapy OR radiotherapy OR radio-resistant OR brachytherapies OR radioresistant OR radio-recurrent OR brachytherapy OR radiorecurrent) AND (prostate OR prostatic) AND (Salvage OR Recurrence OR local failure OR recurrent OR resistant OR radiation failure OR relapse OR recrudescence OR biochemical failure) AND (prostatectomy OR salvage surgery OR prostatectomies OR robot). Additionally, reference lists of eligible studies underwent manual scrutiny for potential additional inclusions.

### 2.3. Study selection and eligibility criteria

#### 2.3.1. Inclusion criteria

Patients diagnosed with radio-recurrent PCa treated with salvage surgery. Availability of quantitative data on either severe complication using Clavien–Dindo scale (CDS) or functional outcomes.

#### 2.3.2. Exclusion criteria

1. Clearly duplicated publications.
2. Articles lacking full-text availability.
3. Publications not written in English.

### 2.4. Data extraction

Two investigators independently employed a standardized data extraction form to collect data, resolving discrepancies through discussion. Patient characteristics data encompassed 2 main aspects: primary treatment features and details of SRP. Additionally, to mitigate the impact of duplicate reports, we meticulously filtered redundant data by considering the institutions of enrollment and enrollment periods, ensuring the accuracy of severe complications and functional outcomes data. The duration of PCa surgery is typically defined as the total operative time, measured from the initiation of the procedure (e.g., skin incision or endoscopic instrument insertion following anesthesia induction) until its conclusion (e.g., wound closure or instrument withdrawal). This interval encompasses all critical surgical steps but excludes ancillary phases such as anesthesia preparation, patient positioning, or postoperative recovery.

### 2.5. Data synthesis and analysis

The primary objective of this study was to evaluate the major complications and functional outcomes in patients with radio-recurrent PCa undergoing salvage surgery, and the secondary objective was to compare the incidence of these 2 categories of events between open surgery and robotic surgery. For this investigation, complications were evaluated using the CDS, where grade ≥ 3a denoted severe events. Functional outcomes included urinary incontinence and erectile dysfunction (ED). Patient was diagnosed incontinent if he needed to use pads, and the urinary incontinence was graded based on the number of pads used by the patient per day, the use of ≥2 pads/d was defined as moderate to severe incontinence, and the use of ≥3 pads/d was defined as severe incontinence. The criterion of postoperative ED was the absence of spontaneous erection with or without phosphodiesterase type 5 inhibitor.

Incidence rates of complications and functional outcomes were summarized along with 95% confidence intervals (CIs). These rates and CIs were calculated using a random effects model with logit transformation, as described by Nyaga et al, and analyzed using STATA 14.0. The odds ratio (OR) for complications and functional outcomes between different subgroups was calculated in the normal fashion, and the statistical significance of the association was assessed using chi-square tests. Statistical significance was determined using a 2-sided test at a significance level (α) of 0.05, with results considered significant if they met this criterion.

## 3. Results

### 3.1. Study selection and patient characteristics

Following removal of duplicate records, a total of 11,253 entries were retrieved from 2 databases. Initial screening of titles and abstracts, and subsequent exclusion of records not meeting inclusion criteria, resulted in retaining a final set of 80 records (see Guidelines Flow Diagram). A comprehensive review of full texts led to the inclusion of 34 studies^[19–52]^ (see Table S1, Supplemental Digital Content, https://links.lww.com/MD/Q152). Following exclusion of 2 nearly completely duplicated studies,^[53,54]^ a summary of severe complications from 28 studies^[19,21,25–31,34–52]^ and functional outcomes from 20 studies^[19,20,22–28,30,32,33,35,39,41,45,47–52]^ was provided, noting partial duplication in 5 studies^[45,47,50–52]^ (see Table S2, Supplemental Digital Content, https://links.lww.com/MD/Q152).

Tables 1 and 2 summarizes primary therapy and the perioperative characteristics of salvage therapy in the included studies. The majority of patients received definitive treatment primarily via external beam radiotherapy (EBRT), supplemented by other modalities such as BT, and proton beam therapy, etc. Across various studies, the median age at recurrence ranged from 61.5 to 76 years, while the median time from primary treatment to salvage therapy varied from 32 to 94.8 months and the median operative time varied from 90 to 303 minutes. Additionally, proportions of neoadjuvant/adjuvant ADT usage and follow-up times after salvage therapy were summarized.

**Table 1:** Primary disease and treatment characteristics.

First author	Year	Design	Patients	Primary treatment
Shiota M	2024	P	10	Carbon ion RT
Yajima S	2023	R	5	RT
Lama DJ	2023	P	78	BT (40%)/EBRT (38%)/PBRT (10%)/BT + EBRT (4%)
Calleris G	2023	R	1030	EBRT (54.8%)/BT (26.2%)/EBRT + BT (2.7%)
Perera M	2022	P	232	RT
Perera M	2022	P	61	RT
Marra G	2022	R	41	RT (82.9%)/BT (4.8%)
Catarino R	2022	NR	29	BT (31.0%)/EBRT (55.2%)/BT + EBRT (3.4%)
Schuetz V	2021	R	53	Irradiation (50.9%)/HDR-BT (1.9%)/LDR-BT (11.3%)/carbon ion irradiation (5.7%)
Ribeiro L	2021	R	90	EBRT (62%)/HDR-BT (4%)/LDR-BT (30%)/cyberknife (1%)/EBRT + BT (2%)
Rajwa P	2021	R	214	EBRT (78%)/BT (18%)/EBRT + BT (3.7%)
Nathan A	2021	R	49	RT (94.1%)/BT (5.9%)
Martinez PF	2021	R	50	BT (14.5%)/3D EBRT (77.6%)/IMRT (7.9%)
Marra G(1)	2021	R	414	EBRT (64.5%)/BT (25.7%)
Marra G	2021	R	414	RT
Madi R	2021	R	20	EBRT (75.0%)/BT (15.0%)
Madi R	2021	R	6	EBRT (50.0%)/BT (16.7%)
Kowalczyk KJ	2021	R	40	EBRT (52.5%)/BT (30.0%)
Kowalczyk KJ	2021	R	32	EBRT (50.0%)/BT (28.1%)/SBRT (6.25%)
Bozkurt Y	2021	R	10	PBT
Bonet X	2021	R	120	RT (75%)
Onol FF	2020	R	94	EBRT (41.5%)/IMRT (16%)/PBRT (3.2%)/BT (24.5%)/EBRT + BT (14.9)
De Groote R	2020	R	106	RT (60%)/BT (22.2%)
Gontero P	2019	R	395	RT (66.84%)/BT (22.28%)
Devos B	2019	R	25	EBRT (68%)/BT (32%)
Ogaya-Pinies G	2018	R	96	EBRT (38.54%)/BT (14.58%)/EBRT + BT (13.54%)/PBRT (1.04%)
Bonet X	2018	R	80	RT (78.7%)
Orré M	2017	NR	7	RSI
Kenney PA	2016	R	20	EBRT or PBRT (65%)/BT or BT + ERBT (35%)
Kenney PA	2016	R	19	EBRT or PBRT (57.8%)/BT or BT + ERBT (42.1%)
Mandel P	2015	R	55	EBRT (49.1%)/HDR-BT (12.7%)/LDR-BT (30.9%)
Yuh B	2014	P	51	BT (43.1%)/BT + EBRT (2.0%)/EBRT (35.3%)/PBRT (11.8%)
Zugor V	2013	R	13	EBRT (53.8%)/BT (46.2%)
Kaffenberger SD	2013	NR	34	BT (38%)/RT (32%)/BT + RT (18%)
Heidenreich A	2010	R	55	RT
Ahallal Y	2010	R	15	EBRT (53.3%)/BT (40%)
Seabra D	2009	P	42	RT
Rogers E	1995	R	40	PL + RSI (26%)/PL + EBRT (3%)/EBRT (27%)

EBRT = external beam radiotherapy, HDR-BT = high-dose-rate brachytherapy, IMRT = intensity-modulated radiation therapy, LDR-BT = low-dose-rate brachytherapy, n = number, NR = not reported, P = prospective, PBRT = proton beam radiation treatment, PL = pelvic lymphadenectomy, R = retrospective, RP = radical prostatectomy, RSI = radioactive seed implantation, RT = radiotherapy, SBRT = stereotactic radiotherapy.

**Table 2:** Disease and treatment characteristics during the peri-salvage surgery period.

First author	Median age at recurrence (yr, range)	Median TRS (mo, range)	ADT usage (%)	Median operative time (min, range)	Surgical method	Pathological GS (%)		Pathological T-stage (%)		Follow-up (mo, range)	Toxicity scale
						≤7	≥8	≤T2	≥T3		
Shiota M	69 (61–74)	NR	Neo-ADT: 50%/Adj-ADT:10%	235 (175–302)	Robot	30	60	40	60	NR	CD
Yajima S	76 (71–77)	75 (61–193)	Neo-ADT: 80%/Adj-ADT: 20%	127 (113–158)	Robot	20	60	40	60	8	CD
Lama DJ	67.0 (63.0–71.0)	67.6 (43.6–96.3)	Neo-ADT: 24%/Adj-ADT: 22%	NR	Robot	54	23	55	45	121 (70–149)	CD
Calleris G	64.9 (60.8–68.0)	50.5 (25.0–80.0)	Neo-ADT: 21.4%	NR	Open (65.5%)/Laparoscopic (0.9%)/Robot (33.5%)	80.9	16.8	57.5	42.5	34 (13–73)	CD
Perera M	65 (60–69)	NR	NR	NR	Open	46.3	29	39	59.5	120	CD
Perera M	67 (62, 71)	NR	NR	NR	Minimally invasive	47.6	44	33	67	120	CD
Marra G	70 (66.5–73)	57 (35.7–118.85)	Neo-ADT: 21.9%/Adj-ADT: 41.4%	240 (175–274)	Open	46.1	39	53.6	46.4	NR	CD
Catarino R	65 (61–68)	53 (6–98)	Neo-ADT: 27.6%	90 (70–150)	Laparoscopic	34.5	44.8	34.4	65.6	94 (7–162)	CD
Schuetz V	68 (64.0–72)	55 (19–74)	Neo-ADT: 26.4%	215 (191–247)	Open (47.2%)/Robot (52.8%)	NR	NR	50.9	49.1	52.6	DP
Ribeiro L	66 (62–70)	NR	NR	NR	Robot (82%)/Laparoscopic (9%)/Open (9%)	62.2	41.1	36	64	29.5 (1.6–139.3)	CD
Rajwa P	69 (64–72)	NR	NR	NR	Open	59.5	40.1	26	74	25.3 (15–28.5)	CD
Nathan A	70 (63–73)	NR	Adj-ADT: 36.7%	160 (125–180)	Robot	73.5	26.5	32.7	67.3	17.1 (10.0–31.3)	CD
Martinez PF	64.5 (60–68)	42 (24–60)	Neo-ADT: 84.3%/Adj-ADT: 39.5%	NR	Open (65.8%)/Robot (34.2%)	31.6	68.4	32.9	67.1	47 (18.5–81)	CD
Marra G	66 (62–70)	62.4 (34.8–90)	NR	186.5 (149–240)	Open (52.2%)/Robot (47.8%)	60.4	39.6	47.1	52.9	36 (20.4–60.5)	CD
Marra G	70 (66–73)	69.4	Neo-ADT: 90%/Adj-ADT: 30%	225 (186–225)	Open (91.3%)/Robot (8.7%)	NR	NR	NR	NR	48	CD
G(1)Madi R	70.0 (64.0–73.0)	94.8 (73.2–160.8)	NR	NR	Retzius-sparing robot	45	55	60	40	18.0 (10.0–26.0)	DP
Madi R	65.5 (57.8–68.0)	94.8 (73.2–160.8)	NR	NR	Standard robot	33.3	66.7	50	50	18.0 (10.0–26.0)	DP
Kowalczyk KJ	68 (63–70)	NR	Adj-ADT: 12.8%	NR	Retzius-sparing robot	55	37.5	50	50	23	CD
Kowalczyk KJ	66 (60–70)	NR	Adj-ADT: 15.6%	NR	Standard robot	50	43.8	31.2	68.8	36	CD
Bozkurt Y	66.8 (57–74)	58.4	NR	230	Robot	10	70	20	80	31.8 (17–65)	CD
Bonet X	65.6	76.4	Adj-ADT: 20%	127	Robot	64.2	35.8	NR	NR	44 ± 26.2	CD
Onol FF	65	82.2	Neo-ADT: 25.5%	128.95	Robot	40.4	42.6	50	50	32 ± 24.21	CD
De Groote R	67	NR	Adj-ADT: 31%	142 (94–210)	Robot	71.1	28.9	33	67	25.2 (19.2–33.6)	DP
Gontero P	66.3 (61.8–70.5)	63.6 (37.2–94.8)	NR	Robot 228 (178–255)/Open 214 (136–240)	Open (47.1%)/Robot (52.9%)	56.57	43.4	45.3	54.71	36 (20.4–58.8)	CD
Devos B	65.0 (52.0–75.6)	NR	Neo-ADT: 36%/Adj-ADT: 0	166.0 (53.5)	Open	40	48	36	64	43	CD
Ogaya-Pinies G	65.8 (61.3–71.1)	81.50 (18.25–335.5)	Adj-ADT: 27.1%	125 (119–138)	Robot	59.4	30.2	49.9	47.9	14 (5–24)	CD
Bonet X	65.6	63.2	Adj-ADT: 15%	128.06	Robot	61.2	38.8	47.5	52.5	22.5 ± 18.1	CD
Orré M	66 (62–71)	46 (26–99)	NR	148	Robot	71.4	28.6	28.6	71.4	24	DP
Kenney PA	66	NR	NR	303.3	Robot	30	45	30	70	16.8	CD
Kenney PA	66	NR	NR	291.5	Open	36.8	47.3	47.3	52.6	16.8	CD
Mandel P	65.4 (49–75)	NR	Adj-ADT: 18.2%	NR	Open	76.4	23.6	50	50	36	CD
Yuh B	68 (63–71)	68 (40–96)	NR	178.8 (144–222)	Robot	60.7	21.6	49	51	36	CD
Zugor V	63.1 (51–74)	48.9 (12–108)	Adj-ADT: 100%	154 (110–200)	Robot	46.2	53.8	NR	NR	22.8 (4–52)	CD
Kaffenberger SD	61–70	48.5	Neo-ADT: 12%	176 (159–191)	Robot	59	26	53	47	16.1 (8.4–31.8)	CD
Heidenreich A	65.3 (45–82)	32 (19–69)	Neo-ADT: 21.8%/Adj-ADT: 0	120 (95–165)	Open	80	20	73.3	26.7	23 (2–56)	CD
Ahallal Y	62.3 (57.4–71.4)	NR	NR	235 (210–285)	Open (73.3%)/Robot (26.7%)	53.3	46.7	40	60	8 (2–17)	CD
Seabra D	61 (59–69)	NR	NR	80 (50–160)	Open	86	14	47	53	18 (1–36)	DP
Rogers E	61.5 (55–73)	58.9 (14–144)	NR	264 (168–420)	Open	NR	NR	20	80	39.3 (2–97)	DP

Adj-ADT = adjuvant androgen deprivation therapy, ADT = androgen deprivation therapy, CD = Clavien–Dindo, DP = descriptive, GS = Gleason score, Neo-ADT = neoadjuvant androgen deprivation therapy, NR = not recorded, TRS = primary treatment to salvage therapy.

### 3.2. Pooled analysis of severe complications according to Clavien–Dindo scale

Overall, 513 (14.64%) of the 3503 patients in the 28 studies had grade 3 or higher complications, and more than 17 different types of severe complications were reported in these 28 studies (Table S3, Supplemental Digital Content, https://links.lww.com/MD/Q152). As shown in Table 3, the common severe complications (CDS grade ≥ 3a) were as follows: urethral stricture (5.90%; 95% CI, 4.23–7.98%), rectourethral/vesicoperineal fistula (1.66%; 95% CI, 0.83–2.96%), hematuria (0.94%; 95% CI, 0.35–2.04%), ureteral injury (0.30%; 95% CI, 0.04–1.09%), myocardial infarction (0.16%; 95% CI, 0.00–0.87%); lymphocele (0.47%; 95% CI, 0.10–1.37%), rectal lesion/injury (1.51%; 95% CI, 0.73–2.76%), ileus (0.47%; 95% CI, 0.10–1.37%), abscess (0.16%; 95% CI, 0.00–0.87%), urinary retention (0.30%; 95% CI, 0.04–1.09%), anastomotic leak (1.37%; 95% CI, 0.59–2.68%), urosepsis (1.42%; 95% CI, 0.65–2.67%), penile/voiding pain (0.17%; 95% CI, 0.00–0.95%), incisional hernia (0.31%; 95% CI, 0.04–1.13%), atrial fibrillation (0.47%; 95% CI, 0.10–1.37%), deep vein thrombosis or pulmonary embolism (0.63%; 95% CI, 0.17–1.60%), urinary tract infection (0.17%; 95% CI, 0.00–0.95%).

**Table 3:** Pooled analysis of functional outcomes and major complications after salvage surgery.

Complications and functional outcomes	Patients (n)	Events (n)	Incidence (%)	95% CI
1-yr incontinence (≥1 pad/d)	1281	635	49.57	46.80–52.35%
1-yr incontinence (≥2 pads/d)	1247	307	24.62	22.25–27.11%
1- yr incontinence (≥3 pads/d)	1031	183	17.75	15.46–20.22%
1- yr any-grade erectile dysfunction	1429	872	61.02	58.44–63.56%
Major complications				
Urethral stricture	661	39	5.90	4.23–7.98%
Rectourethral/vaginoperineal fistula	661	11	1.66	0.83–2.96%
Hematuria	636	6	0.94	0.35–2.04%
Ureteral injury	661	2	0.30	0.04–1.09%
Myocardial infarction	636	1	0.16	0–0.87%
Lymphocele	636	3	0.47	0.10–1.37%
Rectal lesion/injury	661	10	1.51	0.73–2.76%
Ileus	636	3	0.47	0.10–1.37%
Abscess	636	1	0.16	0.00–0.87%
Urinary retention	661	2	0.30	0.04–1.09%
Anastomotic leak	585	8	1.37	0.59–2.68%
Urosepsis	636	9	1.42	0.65–2.67%
Penile/voiding pain	585	1	0.17	0–0.95%
Incisional hernia	636	2	0.31	0.04–1.13%
Atrial fibrillation	636	3	0.47	0.10–1.37%
Deep vein thrombosis or pulmonary embolism	636	4	0.63	0.17–1.60%
Urinary tract infection	585	1	0.17	0–0.95%
Total major complications	3503	513	14.56	13.44–15.74%

### 3.3. Pooled analysis of functional outcomes

Among the 20 studies, 11 and 13 reported the incidence of incontinence ≥ 1 pad/d and incontinence ≥ 2 pads/d after SRP at 1-year, respectively. The overall incidence was 49.57% (635 of 1281) and 24.62% (307 of 1247), respectively (Table S4, Supplemental Digital Content, https://links.lww.com/MD/Q152). In addition, 3 studies reported the incidence of incontinence ≥ 3 pads/d (severe incontinence) after SRP at 1-year (Table S4, Supplemental Digital Content, https://links.lww.com/MD/Q152). The overall incidence of severe incontinence was 17.75% (183 of 1031).

Among the 20 studies, 10 reported the incidence of ED after SRP at 1-year (Table S5, Supplemental Digital Content, https://links.lww.com/MD/Q152). The overall incidence was 73.21% (940 of 1284).

### 3.4. Subgroup analysis of adverse event incidence

#### 3.4.1. Major complications

According to Table S3, Supplemental Digital Content, https://links.lww.com/MD/Q152, 28 papers documented severe complications. Patients with longer operative time (≥150 minutes vs <150 minutes (16.85% vs 3.55%), OR 4.75 (95% CI: 2.92–7.71), *P* < .001) had significantly higher rates of severe complications (see Tables 4 and S6, Supplemental Digital Content, https://links.lww.com/MD/Q152). In terms of surgical methods, there was no significant difference in severe complication rates between the 2 methods (open vs robotic [12.6% vs 11.5%], OR [95% CI], 1.11 [0.76–1.63], *P* = .588; see Tables 5 and S7, Supplemental Digital Content, https://links.lww.com/MD/Q152).

**Table 4:** Comparison of functional outcomes and major complications between different operative time (<150 minutes vs ≥150 minutes).

Complications and functional outcomes	≥150 min			<150 min			≥150 min vs <150 min	
	Patients (n)	Events (n)	Incidence (95% CI)	Patients (n)	Events (n)	Incidence (95% CI)	OR (95% CI)	*P*
1- yr incontinence (≥1 pad/d)	373	256	68.63% (63.66–73.31%)	226	153	67.70% (61.18–73.75%)	1.04 (0.73–1.49)	.812
1- yr incontinence (≥2 pads/d)	459	185	40.31% (35.78–44.95%)	226	61	26.99% (21.32–33.28%)	1.49 (1.17–1.90)	.001
1- yr any-grade erectile dysfunction	247	228	92.31% (88.25–95.31%)	170	156	91.76% (86.57–95.42%)	1.08 (0.52–2.21)	.84
Major complications	1116	188	16.85% (14.69–19.17%)	479	17	3.55% (2.08–5.62%)	4.75 (2.92–7.71)	<.001

CI = confidence interval, n = number, OR = odds ratio.

**Table 5:** Comparison of functional outcomes and major complications between open and robotic surgery.

Complications and functional outcomes	Open			Robotic			Open vs robotic	
	Patients (n)	Events (n)	Incidence (95% CI)	Patients (n)	Events (n)	Incidence (95% CI)	OR (95% CI)	*P*
1- yr incontinence (≥1 pad/d)	225	165	73.33% (67.05–78.99%)	346	222	64.16% (58.86–69.22%)	1.14 (1.02–1.28)	.022
1- yr incontinence (≥2 pads/d)	256	111	43.36% (37.20–49.67%)	446	140	31.39% (27.11–35.92%)	1.38 (1.14–1.68)	.001
1- yr any-grade erectile dysfunction	129	111	86.05% (78.85–91.52%)	277	246	88.81% (84.49–92.27%)	0.78 (0.42–1.45)	.426
Major complications	380	48	12.63% (9.46–16.40%)	712	82	11.52% (9.27–14.09%)	1.11 (0.76–1.63)	.588

CI = confidence interval, n = number, OR = odds ratio.

#### 3.4.2. Urinary incontinence

Initially, the studies by Marra G et al, Schuetz V et al, and Shiota M et al reported significantly higher rates of urinary incontinence than the studies by Calleris G et al, Orré M et al, and Ribeiro L et al (Table S4, Supplemental Digital Content, https://links.lww.com/MD/Q152). In addition, open surgery was associated with a higher incidence of urinary incontinence (≥1 pad/d) compared with robot-assisted surgery (open vs robotic [73.33% vs 64.16%], OR 1.14 [95% CI: 1.02–1.28], *P* = .022; Tables 5 and S8, Supplemental Digital Content, https://links.lww.com/MD/Q152), and the incidence of moderate to severe incontinence (≥2 pads/d) was higher for open surgery (open vs robotic, [43.36% vs 31.39%], OR 1.38 [95% CI: 1.14–1.68], *P* = .001; Table 6 and S9, Supplemental Digital Content, https://links.lww.com/MD/Q152). In contrast, the operative time had no statistically significant effect on the incidence of any-grade urinary incontinence (≥1 pad/d; ≥150 minutes vs <150 minutes, OR 1.04 [95% CI: 0.73–1.49], *P* = .812; Tables 4 and S10, Supplemental Digital Content, https://links.lww.com/MD/Q152). However, patients with a longer operative time had a significantly higher incidence of moderate to severe urinary incontinence (≥150 minutes vs <150 minutes [40.31% vs 26.99%], OR 1.49 [95% CI: 1.17–1.90], *P* < .001; Tables 4 and S11, Supplemental Digital Content, https://links.lww.com/MD/Q152).

#### 3.4.3. Erectile dysfunction

No significant difference in the incidence of 1-year any-grade sexual dysfunction was observed between different surgical approaches (open vs robotic, OR 0.78 [95% CI: 0.42–1.45], *P* = .426; see Table S12, Supplemental Digital Content, https://links.lww.com/MD/Q152 and Table 5) and operative time (≥150 minutes vs <150 minutes, OR 1.08 [95% CI: 0.52–2.21], *P* = .84; see Table S13, Supplemental Digital Content, https://links.lww.com/MD/Q152 and Table 4).

## 4. Discussion

To the best of our knowledge, this meta-analysis assessed the most recent and comprehensive set of severe complications (using CDS) and functional outcomes after salvage surgery in patients with radio-recurrent PCa. Based on a thorough review of current clinical trials, we summarized that the main severe complications that occur in patients with radio-recurrent PCa after salvage surgery include urethral stricture, rectourethral/vesicoperineal fistula, rectal lesion/injury, etc. A high proportion of patients developed urinary incontinence or/and ED, and severe complications were also not rare in the enrolled studies. In balancing the safety and efficacy of salvage surgery, careful consideration should be given to the relatively high incidence of adverse events.

After salvage surgery, urethral stricture (5.90%), rectourethral/vesicoperineal fistula (1.66%), rectal lesion/injury (1.51%), anastomotic leak (1.37%) and urosepsis (1.42%) were common complications. Rectal injury encountered during SRP was probably related to extensive fibrosis and adhesions after RT with subsequently more difficult visualization of the dissection plane between rectum and prostate. For patients with radio-recurrent PCa, the incidence of severe complications from salvage surgery in our study was 14.64%, which is higher than the 5% to 10% reported by Yang J et al for salvage BT and 9% for cryotherapy, but lower than the 22% for salvage high-intensity focused ultrasound.^[55]^ The meta-analysis by Valle LF et al^[11]^ found significantly higher rates of severe genitourinary toxicities with SRP than in this paper (Valle LF vs this paper, 20% vs 14.64%). This may be related to the different included studies. Valle LF et al enrolled available manuscripts through November 19, 2019, whereas half of the articles enrolled in our study were published after the 2020 publication period. Since 2019, there has been a clear increase in robotic surgery compared to conventional open surgery.^[56]^ In addition, our study used the CDS to assess complications, and the CDS did not categorize severe urinary incontinence or ED as serious complications. Given the relatively high incidence of severe urinary incontinence after salvage surgery, it is possible that the rates of serious complications reported in this study are lower than those actually observed in the clinic.

The change in surgical methods (open vs robotic) did not make a significant difference in the incidence of postoperative sexual dysfunction and severe complications. They were similar to those in recently reported, single center or Multicenter results.^[19–21]^ This meta-analysis showed that the overall incidence of severe complications (14.64%) was higher than the incidence in both the open (12.63%) and robotic (11.52%) subgroups. This discrepancy may stem from differences in the studies included. In the enrollment studies of overall incidence of severe complications, Devos B et al (76%, 19 of 25), Marra G et al (50%, 10 of 20) and Kenney PA et al (33%, 13 of 39) reported significantly higher rates of severe complications than others. However, the above studies could not be included in the subgroup study as they did not clearly differentiate between surgical modalities and count the incidence of severe complications separately. Similarly, this meta-analysis found that the overall incidence of ED (73.21%) was lower than that of the open (86.05%) and robotic (88.81%) subgroups, which may also be due to differences in the included studies. Among the enrolled studies, Calleris G et al reported a significantly lower incidence of ED (57.97%, 389 of 671) than the other studies. And the sample size enrolled by Calleris G et al represents approximately 1/2 of the total sample size enrolled (671/1464) in this meta-analysis regarding ED analysis. Similarly, it was not included in the subgroup analysis due to its lack of separate subgroup data.

This meta-analysis showed the incidence of urinary incontinence and moderate to severe incontinence at 1-year in robotic surgery were both approximately 10% lower than open surgery. This was also found in 2 studies.^[19,21]^ Martinez PF et al^[21]^ found that the incidence of incontinence was approximately 25% lower after robotic surgery than after open surgery. Therefore, the ability to maintain continence may represent a significant advantage of robotic surgery compared to untreated ED and severe complications.

This meta-analysis confirms for the first time that shorter operative time is associated with lower rates of severe complications and moderate to severe urinary incontinence. The overall incidence of urinary incontinence was significantly lower than in the operative time subgroups (overall vs <150 minutes vs ≥150 minutes, 49.57% vs 67.70% vs 68.63%). The same is true for moderate to severe incontinence (overall vs <150 minutes vs ≥150 minutes, 24.62% vs 26.99% vs 40.31%). The main reason for this discrepancy is that we enrolled the study of Calleris G et al in the statistics of overall incidence and excluded it from the subgroup analysis due to its lack of operative time. Calleris G et al reported a sample size of more than half of all enrolled studies (749/1281), and their statistical incidence of urinary incontinence (Calleris G vs overall, 33.51% vs 49.57%) and moderate to severe incontinence (Calleris G vs overall, 18.96% vs 24.62%) was significantly lower than the overall incidence.

In subgroup analyses, robotic surgery had a better prognosis in terms of continence compared with open surgery (open vs robotic (incontinence ≥ 1 pad/d, OR 1.14, 95% CI: 1.02–1.28, *P* = .022), (incontinence ≥ 2 pads/d, OR 1.38, 95% CI: 1.14–1.68, *P* = .001), and the shorter operative time reduced the incidence of severe complications (≥150 minutes vs <150 minutes, OR 4.75 [95% CI: 2.92–7.71], *P* < .001). The correlation between operative duration and postoperative infections can be attributed to various time-dependent factors, such as prolonged microbial exposure and diminished efficacy of antimicrobial prophylaxis.^[57,58]^ Postoperative thromboembolism may be associated with factors such as hypercoagulability and endothelial injury caused by prolonged surgical procedures.^[59]^ Prolonged surgical time may also signify increased fatigue among the surgical team and a higher incidence of technical errors. Furthermore, longer operative times often indicate greater procedural complexity.^[60]^ These insights may guide urologists and radiation oncologists to make more confident decisions regarding aggressive treatment.

Our study has several strengths, including the number of studies/patients included, a clear distinction in surgical method, inclusion of up-to-date data, and their careful review of study inclusion. Some limitations must be acknowledged. The main limitation is the significant risk of bias, as most of the included studies were retrospective, which prevented us from reaching a high level of evidence and from providing clear recommendations. In addition, the differences in baseline characteristics of the patients (e.g., initial treatment, surgical approach, age at recurrence, pathologic GS, pathologic T, and primary treatment to salvage therapy) were statistically significant. These differences inevitably influenced the conclusion of toxicity differences between subgroups. Despite our efforts to avoid duplication of data, some studies may overlap, especially from tertiary referral centers in the United States, Australia, and Europe. In addition, as previously mentioned, our study used the CDS to assess complications, whereas incontinence and ED were statistically analyzed separately. Given the relatively high incidence of severe urinary incontinence after salvage surgery, it is possible that the rates of serious complications reported in this study are lower than those actually observed in the clinic. Finally, the functional status of patients before salvage surgery was not reported in our review due to the large number of studies that did not enumerate the functional status of patients before salvage surgery, an important limitation to be aware of. Therefore, the reliability of our findings may be compromised and needs to be validated by relevant randomized controlled trials.

## 5. Conclusions

For patients with recurrent PCa after initial radiotherapy, salvage surgery needs to be used with caution and strict indications. Innovations in surgical methods, such as robotic surgery, along with enhancements in surgical techniques that lead to shorter operative times, appear to reduce the rates of postoperative incontinence and severe complications, respectively. Due to the low level of evidence, relevant RCTs are necessary to confirm this.

## Author contributions

**Conceptualization:** Yachun Zhou, Qing Zhong.

**Data curation:** Xiujun He.

**Methodology:** Yachun Zhou.

**Project administration:** Yachun Zhou.

**Resources:** Yachun Zhou.

**Software:** Yachun Zhou, Qin Yu.

**Supervision:** Xiujun He, Qing Zhong.

**Validation:** Xiujun He, Qing Zhong.

**Visualization:** Xiujun He, Qin Yu.

**Writing – original draft:** Yachun Zhou, Qing Zhong.

**Writing – review & editing:** Qin Yu, Qing Zhong.

## Supplementary Material
