# Supplementary material for: Functional outcomes and complications following salvage radical prostatectomy for post radiotherapy recurrent prostate cancer: A meta-analysis
Source: Medicine (Baltimore). 2025 Sep 26;104(39):e44440. doi: 10.1097/MD.0000000000044440 (PMC13592979; doi:10.1097/MD.0000000000044440)
Supplement: Supplementary file 1 [file medi-104-e44440-s001.pdf]

Supplement table 1 Citation links to the 34 studies enrolled.

ID	Publication year and first author	PMID	Cite
1	2024Shiota M	38212264	Shiota M, Tsukahara S, Takamatsu D, Tanegashima T, Ueda S, Blas L, Goto S, Kobayashi S, Matsumoto T, Inokuchi J, Shioyama Y, Eto M. Salvage robot-assisted radical prostatectomy after carbon ion radiotherapy to the prostate. Asian J Endosc Surg. 2024 Jan;17(1):e13279. doi: 10.1111/ases.13279. PMID: 38212264.
2	2023Yajima S	37324313	Yajima S, Nakanishi Y, Umino Y, Okubo N, Tanabe K, Kataoka M, Masuda H. Salvage Robot-Assisted Radical Prostatectomy (sRARP) for Radiation-Recurrent Prostate Cancer: a Single-Center Experience. Indian J Surg Oncol. 2023 Jun;14(2):361-365. doi: 10.1007/s13193-022-01671-y. Epub 2022 Nov 25. PMID: 37324313; PMCID: PMC10267029.
3	2023Lama DJ	38375345	Lama DJ, Thomas K, Ferenczi B, Okunowo O, Lau CS, Yuh BE. Long-term Outcomes and Patient Satisfaction Following Salvage Robot-assisted Radical Prostatectomy: A Modern Perspective. Eur Urol Open Sci. 2023 Dec 30;60:1-7. doi: 10.1016/j.euros.2023.11.011. PMID: 38375345; PMCID: PMC10874866.
4	2023Calleris G	36682962	Calleris G, Marra G, Benfant N, Rajwa P, Ahmed M, Abreu A, Cacciamani G, Ghoreifi A, Ribeiro L, Westhofen T, Tourinho-Barbosa R, Raskin Y, Veerman H, Albisinni S, Smith JA, Rouprêt M, Oderda M, Massari E, Persad R, Van Der Poel H, Joniau S, Sanchez-Salas R, Kretschmer A, Cathcart P, Gill I, Karnes RJ, Tilki D, Shariat SF, Touijer K, Gontero P. Salvage Radical Prostatectomy for Recurrent Prostate Cancer Following First-line Nonsurgical Treatment: Validation of the European Association of Urology Criteria in a Large, Multicenter, Contemporary Cohort. Eur Urol Focus. 2023 Jul;9(4):645-649. doi: 10.1016/j.euf.2023.01.006. Epub 2023 Jan 20. PMID: 36682962.
5	2022Perera M	35596018	Perera M, Vilaseca A, Tin AL, Nguyen DP, Corradi RB, Touijer AS, Martin-Malburet AG, Alvim R, Benfante N, Sjoberg DD, Laudone V, Scardino PT, Eastham JA, Touijer KA. Morbidity of salvage radical prostatectomy: limited impact of the minimally invasive approach. World J Urol. 2022 Jul;40(7):1637-1644. doi: 10.1007/s00345-022-04031-1. Epub 2022 May 20. PMID: 35596018; PMCID: PMC9357351.
6	2022Marra G	36601045	Marra G, Calleris G, Massari E, Vissio E, Molinaro L, Cassoni P, D'Agate D, Oderda M, Valerio M, Raskin Y, Joniau S, Papotti M, Gontero P. Topography of Prostate Cancer Recurrence: A Single-centre Analysis of Salvage Radical Prostatectomy Specimens and Implications for Focal Salvage Treatments. Eur Urol Open Sci. 2022 Dec 15;47:110-118. doi: 10.1016/j.euros.2022.11.017. PMID: 36601045; PMCID: PMC9806711.
7	2022Catarino R	35591964	Catarino R, Otta-Oshiro RJ, Lista-Mateos F, García-Mediero JM, Nunez-Mora C. Outcomes of laparoscopic salvage radical prostatectomy after primary treatment of prostate cancer. Cent European J Urol. 2022;75(1):59-64. doi: 10.5173/ceju.2022.0271. Epub 2022 Mar 24. PMID: 35591964; PMCID: PMC9074060.
8	2021Schuetz V	35011942	Schuetz V, Reimold P, Goertz M, Hofer L, Dieffenbacher S, Nyarangi-Dix J, Duensing S, Hohenfellner M, Hatiboglu G. Evolution of Salvage Radical Prostatectomy from Open to Robotic and Further to Retzius Sparing Surgery. J Clin Med. 2021 Dec 30;11(1):202. doi: 10.3390/jcm11010202. PMID: 35011942; PMCID: PMC8745561.
9	2021Ribeiro L	35011942	Ribeiro L, Stonier T, Stroman L, Tourinho-Barbosa R, Alghazo O, Winkler M, Dasgupta P, Popert R, Cathelineau X, Sanchez-Salas R, Murphy DG, Emberton M, Cathcart P. Is the Toxicity of Salvage Prostatectomy Related to the Primary Prostate Cancer Therapy Received? J Urol. 2021 Mar;205(3):791-799. doi: 10.1097/JU.0000000000001382. Epub 2020 Oct 6. PMID: 33021441.
10	2021Rajwa P	33997919	Rajwa P, Schuettfort VM, Quhal F, Mori K, Katayama S, Laukhtina E, Pradere B, Motlagh RS, Mostafaei H, Grossmann NC, Aulitzky A, Paradysz A, Karakiewicz PI, Fajkovic H, Zimmermann K, Heidenreich A, Gontero P, Shariat SF. Role of systemic immune-inflammation index in patients treated with salvage radical prostatectomy. World J Urol. 2021 Oct;39(10):3771-3779. doi: 10.1007/s00345-021-03715-4. Epub 2021 May 17. PMID: 33997919; PMCID: PMC8521581.
11	2021Nathan A	33997823	Nathan A, Fricker M, De Groote R, Arora A, Phuah Y, Flora K, Patel S, Kasivisvanathan V, Sridhar A, Shaw G, Kelly J, Briggs T, Rajan P, Sooriakumaran P, Nathan S. Salvage Versus Primary Robot-assisted Radical Prostatectomy: A Propensity-matched Comparative Effectiveness Study from a High-volume Tertiary Centre. Eur Urol Open Sci. 2021 May;27:43-52. doi: 10.1016/j.euros.2021.03.003. PMID: 33997823; PMCID: PMC8090976.
12	2021Martinez PF	33912513	Martinez PF, Romeo A, Tobia I, Isola M, Giudice CR, Villamil WA. Comparing open and robotic salvage radical prostatectomy after radiotherapy: predictors and outcomes. Prostate Int. 2021 Mar;9(1):42-47. doi: 10.1016/j.prnil.2020.07.003. Epub 2020 Aug 2. PMID: 33912513; PMCID: PMC8053694.
13	2021Marra G(1)	33436329	Marra G, Karnes RJ, Calleris G, Oderda M, Alessio P, Palazzetti A, Battaglia A, Pisano F, Munegato S, Munoz F, Filippini C, Ricardi U, Linares E, Sanchez-Salas R, Goonewardene S, Dasgupta P, Challacombe B, Popert R, Cahill D, Gillatt D, Persad R, Palou J, Joniau S, Smelzo S, Piechaud T, Taille A, Roupret M, Albisinni S, van Velthoven R, Morlacco A, Vidit S, Gandaglia G, Mottrie A, Smith J, Joshi S, Fiscus G, Berger A, Aron M, Abreu A, Gill IS, Van Der Poel H, Tilki D, Murphy D, Lawrentschuk N, Davis J, Gontero P. Oncological outcomes of salvage radical prostatectomy for recurrent prostate cancer in the contemporary era: A multicenter retrospective study. Urol Oncol. 2021 May;39(5):296.e21-296.e29. doi: 10.1016/j.urolonc.2020.11.002. Epub 2021 Jan 10. PMID: 33436329.
14	2021Marra G	32414618	Marra G, Calleris G, Alessio P, Oderda M, Palou J, Joniau S, Piechaud T, Smelzo S, Morlacco A, Sharma V, Tilki D, Van der Poel H, Veerman H, Karnes RJ, Gontero P. Outcomes of Salvage Radical Prostatectomy for M0 Castration-resistant Recurrent Prostate Cancer: A Reasonable Option in the Era of New Antiandrogen Therapies? Eur Urol Focus. 2021 Jul;7(4):807-811. doi: 10.1016/j.euf.2020.04.005. Epub 2020 May 12. PMID: 32414618.
15	2021Madi R	33417926	Madi R, Sayyid RK, Hiffa A, Thomas E, Terris MK, Klaassen Z. Early Experience with Salvage Retzius-sparing Robotic-assisted Radical Prostatectomy: Oncologic and Functional Outcomes. Urology. 2021 Mar;149:117-121. doi: 10.1016/j.urology.2020.12.029. Epub 2021 Jan 5. PMID: 33417926.
16	2021Kowalczyk KJ	34181471	Kowalczyk KJ, Madi RH, Eden CG, Sooriakumaran P, Fransis K, Raskin Y, Joniau S, Johnson S, Jacobsohn K, Galfano A, Bocciardi AM, Hwang J, Kim IY, Hu JC. Comparative Outcomes of Salvage Retzius-Sparing versus Standard Robotic Prostatectomy: An International, Multi-Surgeon Series. J Urol. 2021 Nov;206(5):1184-1191. doi: 10.1097/JU.0000000000001939. Epub 2021 Jun 28. PMID: 34181471.
17	2021Bozkurt Y	34462255	Bozkurt Y, Atar M, Pisters LL. Early Experience with Salvage Robotic-Assisted Radical Prostatectomy in Proton Beam Radiotherapy Failures. Balkan Med J. 2021 Sep;38(5):310-315. doi: 10.5152/balkanmedj.2021.21174. PMID: 34462255; PMCID: PMC8880822.
18	2021Bonet X	33256361	Bonet X, Moschovas MC, Onol FF, Bhat KR, Rogers T, Ogaya-Pinies G, Rocco B, Sighinolfi MC, Woodlief T, Vigués F, Patel V. The surgical learning curve for salvage robot-assisted radical prostatectomy: a prospective single-surgeon study. Minerva Urol Nephrol. 2021 Oct;73(5):600-609. doi: 10.23736/S2724-6051.20.04077-1. Epub 2020 Dec 1. PMID: 33256361.
19	2020Onol FF	31430422	Onol FF, Bhat S, Moschovas M, Rogers T, Ganapathi H, Roof S, Rocco B, Patel V. Comparison of outcomes of salvage robot-assisted laparoscopic prostatectomy for post-primary radiation vs focal therapy. BJU Int. 2020 Jan;125(1):103-111. doi: 10.1111/bju.14900. Epub 2019 Oct 3. PMID: 31430422.
20	2020De Groote R	32461073	De Groote R, Nathan A, De Bleser E, Pavan N, Sridhar A, Kelly J, Sooriakumaran P, Briggs T, Nathan S. Techniques and Outcomes of Salvage Robot-Assisted Radical Prostatectomy (sRARP). Eur Urol. 2020 Dec;78(6):885-892. doi: 10.1016/j.eururo.2020.05.003. Epub 2020 May 24. PMID: 32461073.
21	2019Gontero P	31075058	Gontero P, Marra G, Alessio P, Filippini C, Oderda M, Munoz F, Linares E, Sanchez-Salas R, Challacombe B, Dasgupta P, Goonewardene S, Popert R, Cahill D, Gillatt D, Persad R, Palou J, Joniau S, Piechaud T, Morlacco A, Vidit S, Rouprêt M, De La Taille A, Albisinni S, Gandaglia G, Mottrie A, Joshi S, Fiscus G, Berger A, Aron M, Van Der Poel H, Tilki D, Lawrentschuk N, Murphy DG, Leung G, Davis J, Karnes RJ; Collaborators. Salvage Radical Prostatectomy for Recurrent Prostate Cancer: Morbidity and Functional Outcomes from a Large Multicenter Series of Open versus Robotic Approaches. J Urol. 2019 Oct;202(4):725-731. doi: 10.1097/JU.0000000000000327. Epub 2019 Sep 6. PMID: 31075058.
22	2019Devos B	30666400	Devos B, Al Hajj Obeid W, Andrianne C, Diamand R, Peltier A, Everaerts W, Van Poppel H, Van Velthoven R, Joniau S. Salvage high-intensity focused ultrasound versus salvage radical prostatectomy for radiation-recurrent prostate cancer: a comparative study of oncological, functional, and toxicity outcomes. World J Urol. 2019 Aug;37(8):1507-1515. doi: 10.1007/s00345-019-02640-x. Epub 2019 Jan 21. PMID: 30666400.
23	2018Ogaya-Pinies G	30006908	Ogaya-Pinies G, Linares-Espinos E, Hernandez-Cardona E, Jenson C, Cathelineau X, Sanchez-Salas R, Patel V. Salvage robotic-assisted radical prostatectomy: oncologic and functional outcomes from two high-volume institutions. World J Urol. 2019 Aug;37(8):1499-1505. doi: 10.1007/s00345-018-2406-4. Epub 2018 Jul 13. PMID: 30006908.
24	2018Bonet X	30126045	Bonet X, Ogaya-Pinies G, Woodlief T, Hernandez-Cardona E, Ganapathi H, Rogers T, Coelho RF, Rocco B, Vigués F, Patel V. Nerve-sparing in salvage robot-assisted prostatectomy: surgical technique, oncological and functional outcomes at a single high-volume institution. BJU Int. 2018 Nov;122(5):837-844. doi: 10.1111/bju.14517. Epub 2018 Sep 14. PMID: 30126045.
25	2017Orré M	28396223	Orré M, Piéchaud T, Sargos P, Richaud P, Roubaud G, Thomas L. Oncological and functional results of robotic salvage radical prostatectomy after permanent brachytherapy implants. Cancer Radiother. 2017 Apr;21(2):119-123. doi: 10.1016/j.canrad.2016.11.001. Epub 2017 Apr 7. PMID: 28396223.
26	2016Kenney PA	27347619	Kenney PA, Nawaf CB, Mustafa M, Wen S, Wszolek MF, Pettaway CA, Ward JF, Davis JW, Pisters LL. Robotic-assisted laparoscopic versus open salvage radical prostatectomy following radiotherapy. Can J Urol. 2016 Jun;23(3):8271-7. PMID: 27347619; PMCID: PMC5512888.
27	2015Mandel P	25711672	Mandel P, Steuber T, Ahyai S, Kriegmair M, Schiffmann J, Boehm K, Heinzer H, Michl U, Schlomm T, Haese A, Huland H, Graefen M, Tilki D. Salvage radical prostatectomy for recurrent prostate cancer: verification of European Association of Urology guideline criteria. BJU Int. 2016 Jan;117(1):55-61. doi: 10.1111/bju.13103. Epub 2015 May 11. PMID: 25711672.
28	2014Yuh B	24314031	Yuh B, Ruel N, Muldrew S, Mejia R, Novara G, Kawachi M, Wilson T. Complications and outcomes of salvage robot-assisted radical prostatectomy: a single-institution experience. BJU Int. 2014 May;113(5):769-76. doi: 10.1111/bju.12595. PMID: 24314031.
29	2013Zugor V	24008772	Zugor V, Labanaris AP, Porres D, Heidenreich A, Witt JH. Robot-assisted radical prostatectomy for the treatment of radiation-resistant prostate cancer: surgical, oncological and short-term functional outcomes. Urol Int. 2014;92(1):20-6. doi: 10.1159/000351948. Epub 2013 Aug 31. PMID: 24008772.
30	2013Kaffenberger SD	23000849	Kaffenberger SD, Keegan KA, Bansal NK, Morgan TM, Tang DH, Barocas DA, Penson DF, Davis R, Clark PE, Chang SS, Cookson MS, Herrell SD, Smith JA Jr. Salvage robotic assisted laparoscopic radical prostatectomy: a single institution, 5-year experience. J Urol. 2013 Feb;189(2):507-13. doi: 10.1016/j.juro.2012.09.057. Epub 2012 Sep 20. PMID: 23000849; PMCID: PMC3698478.
31	2010Heidenreich A	19303197	Heidenreich A, Richter S, Thüer D, Pfister D. Prognostic parameters, complications, and oncologic and functional outcome of salvage radical prostatectomy for locally recurrent prostate cancer after 21st-century radiotherapy. Eur Urol. 2010 Mar;57(3):437-43. doi: 10.1016/j.eururo.2009.02.041. Epub 2009 Mar 13. PMID: 19303197.
32	2010Ahallal Y	21166755	Ahallal Y, Shariat SF, Chade DC, Mazzola C, Reuter VE, Sandhu JS, Laudone VP, Touijer KA, Guillonneau BD. Pilot study of salvage laparoscopic prostatectomy for the treatment of recurrent prostate cancer. BJU Int. 2011 Sep;108(5):724-8. doi: 10.1111/j.1464-410X.2010.09924.x. Epub 2010 Dec 16. PMID: 21166755; PMCID: PMC4254701.
33	2009Seabra D	19254397	Seabra D, Faria E, Dauster B, Rodrigues G, Fava G. Critical analysis of salvage radical prostatectomy in the management of radioresistant prostate cancer. Int Braz J Urol. 2009 Jan-Feb;35(1):43-8. doi: 10.1590/s1677-55382009000100007. PMID: 19254397.
34	1995Rogers E	7526002	Rogers E, Ohori M, Kassabian VS, Wheeler TM, Scardino PT. Salvage radical prostatectomy: outcome measured by serum prostate specific antigen levels. J Urol. 1995 Jan;153(1):104-10. doi: 10.1097/00005392-199501000-00037. PMID: 7526002.

Supplement table 2 Duplicate studies that were excluded.

Publication year and first author	Surgical method	PMID	Time of enrollment	Institutions of enrollment
2023Marra G	Open (75%)/Robot (25%)	37704503	2000.2-2021.1	14 tertiary referral centers
2018Bonet X	Robot	30126045	2008.1-2016.3	Hospital University de Bellvitge/Global Robotic Institute- Florida Hospital
1998Garzotto M	open	9474190	1985-1993	University of Florida

Supplement table 3 Summary of major complications after salvage RP.

Publication year and first author	Surgical method	Time of enrollment	Institutions of enrollment	Patients (n)	Major complications (n)
2024Shiota M	Robot	2020-2023	Kyushu University Hospital	10	0
2023Yajima S	Robot	2019-2021	National Cancer Center Hospital East	5	1
2023Lama DJ	Robot	2004-2021	City of Hope Comprehensive Cancer Center	78	31
2023Calleris G	Open (65.5%)/Laparoscopic (0.9%)/Robot (33.5%)	2000-2021	14 referral centers	1030	181
2022Perera M	Open (79%)/laparoscopic (21%)/Robot (6%)	1985-2019	Sloan Kettering Cancer Center/Hospital Clinic de Barcelona/Reseau Hospitalier Neuchatelois	293	29
2022Marra G	Open	2007 -2021	Molinette Hospital	41	13
2022Catarino R	Laparoscopic	2007.1-2019.9	Pedro Hispano Hospital	29	3
2021Ribeiro L	Robot (82%)/Laparoscopic (9%)/Open (9%)	2007.4-2018.9	Guys and St Thomas NHS Trust/Kings College Hospital/Institut Mutualiste Montsouris/Sir Peter MacCallum Cancer Centre	90	17
2021Rajwa P	Open	2007-2015	Comprehensive Cancer Center/Medical University of Silesia/University Medical Center Hamburg-Eppendorf/King Fahad Specialist Hospital/The Jikei University School of Medicine	214	26
2021Nathan A	Robot	2012.1-2020.3	University College London Hospitals NHS Foundation Trust	49	1
2021Martinez PF	Open (65.8%)/Robot (34.2%)	2004.8-2019.3	Argentina Hospital Italiano de Buenos Aires	76	7
2021Marra G	Open (91.3%)/Robot (8.7%)	2001-2014	6 institutions	20	10
2021Marra G (1)	Open (52.2%)/Robot (47.8%)	2000-2016	18 tertiary referral centers in United States, Australia and Europe	414	65
2021Kowalczyk KJ	Robot	2011-2020	9 centers	72	10
2021Bozkurt Y	Robot	NR	University of Texas MD Anderson Cancer Center (MDACC)	10	3
2021Bonet X	Robot	2008.7-2018.5	Bellvitge University hospital/advent health global robotics institute/rey Juan Carlos University hospital/University of Modena and reggio emilia	120	3
2020Onol FF	Robot	2008-2018	Advent Health Global Robotics Institute/University of Florida/University of Modena and Reggio Emilia	94	4
2019Gontero P	Open (52.9%)/Robot (47.1%)	2000-2016	18 tertiary referral centers	395	39
2019Devos B	Open (92%)/Robot (8%)	1998-2016	Belgium University Hospitals Leuven/Institute Jules Bordet Brussels	25	19
2018Bonet X	Robot	2008.1-2016.3	Hospital Universitari de Bellvitge/Global Robot Institute–Florida Hospital	80	0
2018Ogaya-Pinies G	Robot	2001.1-2016.4	GRI/Montsouris	96	4
2016Kenney PA	Open (48.7%)/Robot (51.3%)	2007-2011	Yale School/Najaj University/West Virginia University/Massachusetts General Hospital/MD Anderson Cancer Center	39	13
2015Mandel P	Open	2007.1-2012.12	Martini-Clinic Prostate Cancer Center	55	7
2014Yuh B	Robot	2004-2012	City of Hope National Cancer Center/University of	51	22
2013Zugor V	Robot	2006.2-2011.7	Prostate Center Northwest/St. Antonius Hospital/RWTH Aachen University Hospital/Interbalkan Medical Center	13	2
2013Kaffenberger SD	Robot	2006-2011	Vanderbilt University	34	1
2010Heidenreich A	Open	2004.1-2008.8	University of Cologne/RWTH University Aachen	55	2
2010Ahallal Y	Open	2004-2010	Memorial Sloan-Kettering Cancer Center	15	0

continue

Publication year and first author	Patients (n)	US (n)	RF/VF (n)	Hematuria (n)	UI (n)	MI (n)	Lymphocele (n)	RL/RI (n)	Ileus (n)	Abscess (n)	UR (n)	AL (n)	Urosepsis (n)	PP/VP (n)	IH (n)	AF (n)	DVT/PE (n)	UTI (n)
2024Shiota M	10	0	0	0	0	0	0	0	0	0	0	0	0	0	0	0	0	0
2023Yajima S	5	0	0	1	0	0	0	0	0	0	0	0	0	0	0	0	0	0
2023Lama DJ	78	18	1	1	0	0	0	1	1	0	1	0	2	0	1	0	2	1
2023Calleris G	1030	NR	NR	NR	NR	NR	NR	NR	NR	NR	NR	NR	NR	NR	NR	NR	NR	NR
2022Perera M	293	NR	NR	NR	NR	NR	NR	NR	NR	NR	NR	NR	NR	NR	NR	NR	NR	NR
2022Marra G	41	NR	NR	NR	NR	NR	NR	NR	NR	NR	NR	NR	NR	NR	NR	NR	NR	NR
2022Catarino R	29	2	1	0	0	0	0	0	0	0	0	0	0	0	0	0	0	0
2021Ribeiro L	90	NR	NR	NR	NR	NR	NR	NR	NR	NR	NR	NR	NR	NR	NR	NR	NR	NR
2021Rajwa P	214	NR	NR	NR	NR	NR	NR	NR	NR	NR	NR	NR	NR	NR	NR	NR	NR	NR
2021Nathan A	49	NR	NR	NR	NR	NR	NR	NR	NR	NR	NR	NR	NR	NR	NR	NR	NR	NR
2021Martinez PF	76	0	0	1	0	0	0	0	0	0	0	0	4	0	0	2	0	0
2021Marra G	20	NR	NR	NR	NR	NR	NR	NR	NR	NR	NR	NR	NR	NR	NR	NR	NR	NR
2021Marra G(1)	414	NR	NR	NR	NR	NR	NR	NR	NR	NR	NR	NR	NR	NR	NR	NR	NR	NR
2021Kowalczyk KJ	72	NR	NR	NR	NR	NR	NR	NR	NR	NR	NR	NR	NR	NR	NR	NR	NR	NR
2021Bozkurt Y	10	NR	NR	NR	NR	NR	NR	NR	NR	NR	NR	NR	NR	NR	NR	NR	NR	NR
2021Bonet X	120	NR	NR	NR	NR	NR	NR	NR	NR	NR	NR	NR	NR	NR	NR	NR	NR	NR
2020Onol FF	94	NR	NR	NR	NR	NR	NR	NR	NR	NR	NR	NR	NR	NR	NR	NR	NR	NR
2019Gontero P	395	NR	NR	NR	NR	NR	NR	NR	NR	NR	NR	NR	NR	NR	NR	NR	NR	NR
2019Devos B	25	8	4	NR	1	NR	NR	4	NR	NR	0	NR	NR	NR	NR	NR	NR	NR
2018Bonet X	80	0	0	0	0	0	0	0	0	0	0	0	0	0	0	0	0	0
2018Ogaya-Pinies G	96	0	0	0	0	1	1	0	0	0	0	2	0	0	0	0	0	0
2016Kenney PA	39	0	1	2	0	0	1	2	0	0	0	4	1	1	0	0	0	0
2015Mandel P	55	1	3	0	0	0	1	0	1	1	0	0	0	0	0	0	0	0
2014Yuh B	51	10	1	1	1	0	0	0	1	0	1	NR	2	NR	1	1	2	NR
2013Zugor V	13	0	0	0	0	0	0	0	0	0	0	2	0	0	0	0	0	0
2013Kaffenberger SD	34	0	0	0	0	0	0	1	0	0	0	0	0	0	0	0	0	0
2010Heidenreich A	55	0	0	0	0	0	0	2	0	0	0	0	0	0	0	0	0	0
2010Ahallal Y	15	0	0	0	0	0	0	0	0	0	0	0	0	0	0	0	0	0

US urethral stricture, RF/VF Rectourethral/Vaginoperineal fistula, UI Ureteral injury, MI Myocardial infarction, RL/RI Rectal lesion/injury, UR Urinary retention, AL Anastomotic leak, PP/VP Penile/Voiding pain, IH Incisional hernia, AF Atrial fibrillation, DVT/PE Deep vein thrombosis/Pulmonary embolism, UTI Urinary tract infection

Supplementary table 4 Summary of incontinence after salvage RP 1 year.

Publication year and first author	Surgical method	PMID	Time of enrollment	Institutions of enrollment	Patients (n)	Incontinence (1 year), ≥1 pad/d	Incidence (95% CI), ≥1 pad/d	Incontinence (1 year), ≥2 pads/d	Incidence (95% CI),≥ 2 pads/d	Incontinence (1 year), ≥3 pads/d	Incidence (95% CI), ≥3 pads/d
2024Shiota M	Robot	38212264	2020-2023	Kyushu University Hospital	6	NR	NR	4	66.67% (22.28-95.67%)	NR	NR
2023Calleris G	Open (65.5%)/ Laparoscopic (0.9%)/Robot (33.5%)	36682962	2000-2021	14 tertiary referral centers	749	251	33.51% (30.13-37.02%)	142	18.96% (16.21-21.95%)	100	13.35% (11.00-16.00%)
2022Perera M	Open (79%)/ Laparoscopic (15%)/Robot (6%)	35596018	1985-2019	Memorial Sloan Kettering Cancer Center/Hospital Clinic de Barcelona/Reseau Hospitalier Neuchatelois	165	138	83.64% (77.09-88.93%)	NR	NR	NR	NR
2022Marra G	open	36601045	2007 -2021	Molinette Hospital	25	25	100% (86.28-100%)	15	60.00% (38.67-78.87%)	NR	NR
2022Catarino R	Laparoscopic	35591964	2007.1-2019.9	Pedro Hispano Hospital	28	22	78.57% (59.05-91.70%)	NR	NR	10	35.71% (18.64-55.93%)
2021Schuetz V	Robot	35011942	2010—2020	University Hospital Heidelberg	28	NR	NR	18	64.29% (44.07-81.36%)	NR	NR
2021Schuetz V	Open	35011942	2010—2020	University Hospital Heidelberg	25	22	88.00% (68.78-97.45%)	17	68.00% (46.50-85.05%)	NR	NR
2021Ribeiro L	Open (9%)/Laparoscopic (9%)/Robot (82%)	33021441	2007.4-2018.9	Guy’s Hospital/Institut Mutualiste Montsouris/Imperial College Healthcare Trust/The Peter MacCallum Cancer Centre	78	40	51.28% (39.69-62.77%)	5	6.41% (2.11-14.33%)	NR	NR
2021Nathan A	Robot	33997823	2012.1-2020.3	University College London Hospitals NHS Foundation Trust	49	NR	NR	19	38.78% (25.20-53.76%)	NR	NR
2021Madi R	Robot	33417926	2012.1-2020.5	Augusta University Health	17	NR	NR	5	29.41% (10.31-55.96%)	NR	NR
2021Bonet X	Robot	33256361	2008.7-2018.5	Bellvitge University hospital/advent health global robotics institute/rey Juan Carlos University hospital/University of Modena and reggio emilia	120	68	56.67% (47.31-65.68%)	29	24.17% (16.82-32.83%)	NR	NR
2020De Groote R	Robot	32461073	2012.1-2018.12	Onze Lieve Vrouw Hospital Aalst	45	36	80.00% (65.40-90.42%)	20	44.44% (29.64-60.00%)	NR	NR
2019Gontero P	Robot	31075058	2000-2016	18 tertiary institutions	162	110	67.90% (60.12-75.01%)	41	25.31% (18.81-32.73%)	32	19.75% (13.92-26.73%)
2019Gontero P	Open	31075058	2000-2016	18 tertiary institutions	120	93	77.50% (68.98-84.62%)	50	41.67% (32.74-51.02%)	41	34.17% (25.76-43.38%)
2017Orré M	Robot	28396223	2007.10-2012.3	Institut Bergonié	6	2	33.33% (4.33-77.72%)	1	16.67% (0.42-64.12%)	NR	NR
2013Zugor V	Robot	24008772	2006.2-2011.7	Prostate Center Northwest/St. Antonius Hospital/RWTH Aachen University Hospital/Interbalkan Medical Center	13	6	46.15% (19.22-74.87%)	3	23.08% (5.04-53.81%)	NR	NR
2010Heidenreich A	Open	19303197	2004.1-2008.8	Germany University of Cologne/RWTH University Aachen	55	25	45.45% (31.97-59.45%)	11	20.00% (10.43-32.97%)	NR	NR
1995Rogers E	Open	7526002	1984-1992	Baylor College of Medicin/The Methodist Hospital	31	NR	NR	18	58.06% (39.08-75.45%)	NR	NR

Supplementary table 5 Summary of erectile dysfunction after salvage RP 1 year.

Publication year and first author	Surgical method	PMID	Time of enrollment	Institutions of enrollment	Patients (n)	Erectile dysfunction (1 year)	Incidence (95% CI)
2023Yajima S	Robot	37324313	2019-2021	National Cancer Center Hospital East	5	5	100% (47.82-100%)
2023Calleris G	Open (65.5%)/Laparoscopic (0.9%)/Robot (33.5%)	36682962	2000-2021	14 tertiary referral centers	671	389	57.97% (54.14-61.74%)
2022Perera M	Open (79%)/Laparoscopic (15%)/Robot (6%)	35596018	1985-2019	Memorial Sloan Kettering Cancer Center/Hospital Clínic de Barcelona/Reseau Hospitalier Neuchatelois	124	114	91.94% (85.67-96.06%)
2022Catarino R	Laparoscopic	35591964	2007.1-2019.9	Pedro Hispano Hospital	7	6	85.71% (42.13-99.64%)
2021Kowalczyk KJ	Robot	34181471	2011-2020	9 centers	72	64	88.89% (79.28-95.08%)
2021Bonet X	Robot	33256361	2008.7-2018.5	Bellvitge University hospital/advent health global robotics institute/rey Juan Carlos University hospital/University of Modena and reggio emilia	120	101	84.17% (76.38-90.19%)
2020De Groote R	Robot	32461073	2012.1-2018.12	Onze Lieve Vrouw Hospital Aalst	45	44	97.78% (88.23-99.94%)
2019Gontero P	Robot	31075058	2000-2016	18 tertiary institutions	99	91	91.92% (84.70-96.45%)
2019Gontero P	Open	31075058	2000-2016	18 tertiary institutions	86	79	91.86% (83.95-96.66%)
2013Zugor V	Robot	24008772	2006.2-2011.7	Prostate Center Northwest/St. Antonius Hospital/RWTH Aachen University Hospital/Interbalkan Medical Center	13	10	76.92% (46.19-94.96%)
2009Seabra D	Open	19254397	2005.1-2007.6	Pio XII Foundation	43	32	74.42% (58.83-86.48%)

Supplementary table 6 Major complications between open and robotic surgery.

Publication year and first author	Surgical method	Patients(n)	Major complications(n)	Incidence (95% CI)
2010Ahallal Y	Open	15	0	0% (0-21.80%)
2010Heidenreich A	Open	55	2	3.64% (0.44-12.53%)
2015Mandel P	Open	55	7	12.73(5.27-24.48%)
2021Rajwa P	Open	214	26	12.15% (8.09-17.29%)
2022Marra G	Open	41	13	31.71% (18.08-48.09%)
Total		380	48	12.63% (9.46-16.40%)
2013Kaffenberger SD	Robot	34	1	2.94% (0.07-15.33%)
2013Zugor V	Robot	13	2	15.38% (1.92-45.45%)
2014Yuh B	Robot	51	22	43.14% (29.35-57.75%)
2018Bonet X	Robot	80	0	0% (0-4.51%)
2018Ogaya-Pinies G	Robot	96	4	4.17% (1.15-10.33%)
2020Onol FF	Robot	94	4	4.26% (1.17-10.54%)
2021Bonet X	Robot	120	3	2.50% (0.52-7.13%)
2021Bozkurt Y	Robot	10	3	30.00% (6.67-65.25%)
2021Kowalczyk KJ	Robot	72	10	13.89% (6.87-24.06%)
2021Nathan A	Robot	49	1	2.04% (0.05-10.85%)
2023Lama DJ	Robot	78	31	39.74% (28.83-51.46%)
2023Yajima S	Robot	5	1	20.00% (0.51-71.64%)
2024Shiota M	Robot	10	0	0% (0-30.85%)
Total		712	82	11.52% (9.27-14.09%)

Supplementary table 7 Major complications between different operative time (≥150min vs. <150min).

Publication year and first author	Operative time (min, range)	Patients (n)	Major complications (n)	Incidence (95% CI)
2022Catarino R	90 (70–150)	29	3	10.34% (2.19-27.35%)
2010Heidenreich A	120 (95–165)	55	2	3.64% (0.44-12.53%)
2018Ogaya-Pinies G	125 (119–138)	96	4	4.17% (1.15-10.33%)
2023Yajima S	127(113–158)	5	1	20.00% (0.51-71.64%)
2021Bonet X	127±20	120	3	2.50% (0.52-7.13%)
2018Bonet X	128.06 ± 16.1	80	0	0% (0-4.51%)
2020Onol FF	128.95±19.33	94	4	4.26% (1.17-10.54%)
Total	<150min	479	17	3.55% (2.08-5.62%)
2013Zugor V	154 (110–200)	13	2	15.38% (1.92-45.45%)
2021Nathan A	160 (125-180)	49	1	2.04% (0.05-10.85%)
2019Devos B	166.0 (53.5)	25	19	76.00% (54.87-90.64%)
2013Kaffenberger SD	176 (159–191)	34	1	2.94% (0.07-15.33%)
2014Yuh B	178.8 (144–222)	51	22	43.14% (29.35-57.75%)
2021Marra G (1)	186.5 (149−240)	414	65	15.70% (12.33-9.57%)
2019Gontero P	Robot 228(178-255)/ Open 214(136-240)	395	39	9.87% (7.12-13.25%)
2021Marra G	225 (186–225)	20	10	50.00% (27.20-72.80%)
2021Bozkurt Y	230	10	3	30.00% (6.67-65.25%)
2010Ahallal Y	235 (210–285)	15	0	0% (0-21.80%)
2024Shiota M	235(175–302)	10	0	0% (0-30.85%)
2022Marra G	240 (175-274)	41	13	31.71% (18.08-48.09%)
2016Kenney PA	291.5-303.3	39	13	33.33% (19.09-50.22%)
Total	⩾150min	1116	188	16.85% (14.69-19.17%)

Supplementary table 8 Comparison of 1-year incontinence (≥1 pad/d) rates between open and robotic surgery.

Publication year and first author	Surgical method	Patients (n)	Incontinence (1 year), ≥1 pad/d	Incidence (95% CI)
2013Zugor V	Robot	13	6	46.15% (19.22-74.87%)
2021Bonet X	Robot	120	68	56.67% (47.31-65.68%)
2020De Groote R	Robot	45	36	80.00% (65.40-90.42%)
2019Gontero P	Robot	162	110	67.90% (60.12-75.01%)
2017Orré M	Robot	6	2	33.33% (4.33-77.72%)
Total		346	222	64.16% (58.86-69.22%)
2021Schuetz V	Open	25	22	88.00% (68.78-97.45%)
2022Marra G	Open	25	25	100% (86.28-100%)
2010Heidenreich A	Open	55	25	45.45% (31.97-59.45%)
2019Gontero P	Open	120	93	77.50% (68.98-84.62%)
Total		225	165	73.33% (67.05-78.99%)

Supplementary table 9 Comparison of 1-year incontinence (≥2 pads/d) rates between open and robotic surgery.

Publication year and first author	Surgical method	Patients(n)	Incontinence (1 year), ≥2 pads/d	Incidence (95% CI)
2021Madi R	Robot	17	5	29.41% (10.31-55.96%)
2024Shiota M	Robot	6	4	66.67% (22.28-95.67%)
2021Schuetz V	Robot	28	18	64.29% (44.07-81.36%)
2021Nathan A	Robot	49	19	38.78% (25.20-53.76%)
2021Bonet X	Robot	120	29	24.17% (16.82-32.83%)
2020De Groote R	Robot	45	20	44.44% (29.64-60.00%)
2013Zugor V	Robot	13	3	23.08% (5.04-53.81%)
2019Gontero P	Robot	162	41	25.31% (18.81-32.73%)
2017Orré M	Robot	6	1	16.67% (0.42-64.12%)
Total		446	140	31.39% (27.11-35.92%)
2022Marra G	Open	25	15	60.00% (38.67-78.87%)
2021Schuetz V	Open	25	17	68.00% (46.50-85.05%)
2010Heidenreich A	Open	55	11	20.00% (10.43-32.97%)
1995Rogers E	Open	31	18	58.06% (39.08-75.45%)
2019Gontero P	Open	120	50	41.67% (32.74-51.02%)
Total		256	111	43.36% (37.20-49.67%)

Supplementary table 10 Comparison of 1-year incontinence (≥1 pad/d) rates between different operative time (≥150min vs. <150min).

Publication year and first author	Operative time (min, range)	Patients (n)	Incontinence (1 year), ≥1 pad/d	Incidence (95% CI)
2022Catarino R	90 (70–150)	28	22	78.57% (59.05-91.70%)
2010Heidenreich A	120 (95–165)	55	25	45.45% (31.97-59.45%)
2021Bonet X	127±20	120	68	56.67% (47.31-65.68%)
2020De Groote R	142 (94-210)	45	36	80.00% (65.40-90.42%)
2017Orré M	148	6	2	33.33% (4.33-77.72%)
Total	<150min	226	153	67.70% (61.18-73.75%)
2013Zugor V	154 (110–200)	13	6	46.15% (19.22-74.87%)
2021Schuetz V	215 (191–247)	53	22	41.51% (28.14-55.87%)
2019Gontero P	Robot 228(178-255)/ Open 214(136-240)	282	203	71.99% (66.35-77.15%)
2022Marra G	240 (175-274)	25	25	100% (86.28-100%)
Total	⩾150min	373	256	68.63% (63.66-73.31%)

Supplementary table 11 Comparison of 1-year incontinence (≥2 pads/d) rates between different operative time (≥150min vs.<150min).

Publication year and first author	Operative time (min, range)	Patients(n)	Incontinence (1 year), ⩾2 pads/d	Incidence (95% CI)
2010Heidenreich A	120 (95–165)	55	11	20.00% (10.43-32.97%)
2021Bonet X	127±20	120	29	24.17% (16.82-32.83%)
2020De Groote R	142 (94-210)	45	20	44.44% (29.64-60.00%)
2017Orré M	148	6	1	16.67% (0.42-64.12%)
Total	<150min	226	61	26.99% (21.32-33.28%)
2013Zugor V	154 (110–200)	13	3	23.08% (5.04-53.81%)
2021Nathan A	160 (125-180)	49	19	38.78% (25.20-53.76%)
2021Schuetz V	215 (191–247)	53	35	66.04% (51.73-78.48%)
2019Gontero P	Robot 228(178-255)/ Open 214(136-240)	282	91	32.27% (26.85-38.07%)
2024Shiota M	235(175–302)	6	4	66.67% (22.28-95.67%)
2022Marra G	240 (175-274)	25	15	60.00% (38.67-78.87%)
1995Rogers E	264 (168-420)	31	18	58.06% (39.08-75.45%)
Total	⩾150min	459	185	40.31% (35.78-44.95%)

Supplementary table 12 Comparison of 1-year erectile dysfunction rates between open and robotic surgery.

Publication year and first author	Surgical method	Patients (n)	Erectile dysfunction (1 year)	Incidence (95% CI)
2013Zugor V	Robot	13	10	76.92% (46.19-94.96%)
2021Bonet X	Robot	120	101	84.17% (76.38-90.19%)
2020De Groote R	Robot	45	44	97.78% (88.23-99.94%)
2019Gontero P	Robot	99	91	91.92% (84.70-96.45%)
Total		277	246	88.83% (85.04-91.93%)
2009Seabra D	Open	43	32	74.42% (58.83-86.48%)
2019Gontero P	Open	86	79	91.86% (83.95-96.66%)
Total		129	111	86.05% (78.85-91.52%)

Supplementary table 13 Comparison of 1-year erectile dysfunction rates between different operative time (≥150min vs. <150min).

Publication year and first author	Operative time (min, range)	patients(n)	Erectile dysfunction (1 year)	Incidence (95% CI)
2022Catarino R	90 (70–150)	7	6	85.71% (42.13-99.64%)
2023Yajima S	127(113–158)	5	5	100% (47.82-100%)
2021Bonet X	127±20	120	101	84.17% (76.38-90.19%)
2020De Groote R	142 (94-210)	45	44	97.78% (88.23-99.94%)
Total	<150min	170	156	91.76% (86.57-95.42%)
2013Zugor V	154 (110–200)	13	10	76.92% (46.19-94.96%)
2021Nathan A	160 (125-180)	49	48	97.96% (89.15-99.95%)
2019Gontero P	Robot 228(178-255)/ Open 214(136-240)	185	170	91.89% (86.98-95.39%)
Total	⩾150min	247	228	92.31% (88.25-95.31%)
